# Gut Microbiota Changes Following Short-Term Probiotic Supplementation in Older Home Enteral Nutrition Patients

**DOI:** 10.3390/nu18061013

**Published:** 2026-03-23

**Authors:** Niki Tombolesi, Emanuele Francini, Giulia Matacchione, Debora Sparvoli, Nikolina Jukic Peladic, Maurizio Cardelli, Rina Recchioni, Matilde Sbriscia, Sonia Fantone, Chiara Giordani, Angelica Giuliani, Stefania Silvi, Dennis Fiorini, Sabrina Donati Zeppa, Antonio Domenico Procopio, Fabiola Olivieri, Fabrizia Lattanzio, Maria Capalbo, Paolo Orlandoni, Francesca Marchegiani

**Affiliations:** 1 Department of Biomedical Sciences and Public Health, Marche Polytechnic University, Marche University Hospital, 60121 Ancona, Italy; niki.tombolesi@ospedaliriuniti.marche.it; 2 Clinic of Laboratory and Precision Medicine, IRCCS INRCA, 60121 Ancona, Italy; e.francini@inrca.it (E.F.); r.recchioni@inrca.it (R.R.); c.giordani@inrca.it (C.G.); a.giuliani@inrca.it (A.G.); a.d.procopio@univpm.it (A.D.P.); fr.marchegiani@inrca.it (F.M.); 3 Clinical Nutrition, IRCCS INRCA, 60127 Ancona, Italy; d.sparvoli@inrca.it (D.S.); n.jukicpeladic@inrca.it (N.J.P.); p.orlandoni@inrca.it (P.O.); 4 Advanced Technology Center for Aging Research, IRCCS INRCA, 60121 Ancona, Italy; m.cardelli@inrca.it (M.C.); m.sbriscia@inrca.it (M.S.); s.fantone@inrca.it (S.F.); 5 Laboratory of Experimental Pathology, Department of Clinical and Molecular Sciences, Università Politecnica delle Marche, 60100 Ancona, Italy; 6 School of Biosciences and Veterinary Medicine, University of Camerino, Via Gentile III da Varano, 62032 Camerino, Italy; stefania.silvi@unicam.it; 7 School of Science and Technology, Chemistry Division, ChIP (Chemistry Interdisciplinary Project), University of Camerino, Via Madonna delle Carceri, 62032 Camerino, Italy; dennis.fiorini@unicam.it; 8 Department of Biomolecular Sciences, University of Urbino Carlo Bo, 61029 Urbino, Italy; sabrina.donatizeppa@uniroma5.it; 9 Department of Promotion of Human Sciences and Quality of Life, San Raffaele University, 00166 Rome, Italy; 10 Scientific Direction, IRCCS INRCA, 60124 Ancona, Italy; direzionescientifica@inrca.it; 11 General Direction, IRCCS INRCA, 60124 Ancona, Italy; m.capalbo@inrca.it

**Keywords:** probiotics, home enteral nutrition (HEN), gut microbiota, older patients, inflammation, dysbiosis

## Abstract

Background: Home Enteral Nutrition (HEN) patients, often older adults, are susceptible to gut microbiota dysbiosis and low-grade chronic inflammation (inflammaging), which negatively impacts overall health and intestinal integrity. However, evidence on microbiota-targeted interventions in this population remains limited. The development of targeted nutritional strategies, such as probiotic supplementation, has been proposed to address these age-related changes. Methods: This exploratory randomized, open-label study explored changes in gut microbiota composition following a 30-day probiotic intervention in a cohort of sixteen older HEN patients. Gut microbiota profiles were analyzed at baseline and post-intervention using 16S rRNA gene amplicon sequencing. Results: Significant shifts in the gut microbiota were observed, including a statistically significant increase in alpha diversity after 30 days. At the taxonomic level, the treated group showed an increased relative abundance of Lachnospiraceae and Erysipelotrichaceae, suggesting a modulation of gut microbiota structure following probiotic supplementation. Conclusions: These findings provide preliminary insights into microbiota dynamics in this population and may inform the design of future studies integrating functional and clinical outcomes.

## 1. Introduction

Population aging is accelerating in Italy, reflecting a broader demographic trend observed across most Western countries. As is widely recognized, aging is accompanied by a decline in both innate and adaptive immune responses [1,2] a phenomenon called immunosenescence, which is further modulated by nutritional status [3].

Older individuals frequently encounter challenges with deglutition and maintaining a nutritionally adequate diet, significantly compromising their quality of life. In severe instances, patients incapable of self-feeding necessitate enteral nutrition (EN). EN is crucial for providing sustenance to patients unable to consume food orally or fulfill their dietary requirements [4]. Home enteral nutrition (HEN) has therefore become a widely adopted approach for delivering long-term nutritional support in non-hospital settings whenever feasible [5].

Our group previously reported that structured protocols for percutaneous endoscopic gastrostomy (PEG) and HEN can significantly improve the safety and outcomes of long-term nutritional support for multimorbid geriatric patients, thereby mitigating complications and mortality [6]. From a physiological perspective, EN in older patients could modulate the chronic low-grade inflammation associated with ageing, named “inflammaging”, influencing immune function, metabolic homeostasis, and gut–microbiota interaction.

Several laboratory biomarkers have been proposed to support the identification and monitoring of malnutrition in older adults, including inflammatory and metabolic parameters associated with systemic alterations in nutritional status [7]. Indeed, oxidative stress and adipokine dysregulation have been increasingly recognized as important components linking nutritional disturbances with systemic inflammation in aging populations [8].

As a targeted dietary intervention, long-term HEN can also influence the composition and function of the gut microbiota. Enteral formulas often provide limited fermentable substrates, which may reduce microbial diversity and short-chain fatty acid production, thereby promoting gut microbiota dysbiosis. In this setting, strategies aimed to modulating the gut microbiota, such as probiotic supplementation, may represent a rational adjunct to standard EN [9,10].

The gut microbiota, which consists of bacteria, archaea, viruses and fungi, plays a central role in host physiology. This includes nutrient metabolism, modulation of the immune system, and defense against pathogens [11,12,13]. Changes in gut microbiota composition have been linked to various diseases [14]. In frail older people, the structure of the gut microbiota is characterized by reduced biodiversity [15,16]. Such a reduction is commonly associated with dysbiosis, which may further negatively impact the health of these vulnerable older individuals. Indeed, lower diversity is an established risk factor for systemic inflammation, frailty, and increased susceptibility to infections among older adults [17,18,19,20,21]. Interestingly, the gut microbiota of centenarians is characterized by a unique bacterial structure and greater biodiversity [22].

Research has explored the interplay between EN and the microbiota, specifically investigating their reciprocal actions and the ensuing physiological effects [23,24,25]. People who were fed through EN often experience alterations to intestinal transit due to poor intestinal motility. This requires the administration of pre-digested, oligomeric mixtures that are more easily absorbed by the intestine [26,27]. On the one hand, these mixtures are generally better tolerated by patients. However, their fiber-free composition means they lack the substrates necessary to nourish the beneficial bacteria, such as Firmicutes (e.g., *Lactobacillus* and *Bifidobacterium*) that are essential for producing short-chain fatty acids (SCFAs) [28]. SCFAs, particularly butyrate, play a key role in maintaining gut health, and their deficiency has consequently been associated with increased intestinal barrier fragility [29]. Disturbance of the gut microbiota promotes an ecological niche supportive of *Clostridioides difficile* growth [11,26].

In this context, experimental and clinical studies have explored the potential benefits of probiotic supplementation during EN. In a rat model, Zheng et al. showed that short-peptide EN combined with *Lactobacillus acidophilus* improved intestinal barrier integrity, reduced histological injury, and promoted favorable shifts in fecal microbiota compared with EN alone [30]. Clinical studies have also reported potential benefits: Naslowski et al. conducted a randomized, placebo-controlled trial demonstrating effects of probiotic supplementation on infection rates and gastrointestinal parameters in adults receiving EN [31]. In addition, supplementation with *Bifidobacterium longum* BB536 has been shown to increase intestinal bifidobacteria and support immune function in older individuals receiving EN [32,33]. Symbiotic enteral formulas containing *Lactobacillus* and *Bifidobacterium* species have also been investigated in critically ill patients, showing potential benefits for enteral feeding tolerance and nutritional outcomes [34]. Overall, these findings suggest that combining probiotics with EN may help modulate gut microbiota and gastrointestinal function in clinical populations [35].

The growing scientific interest in using probiotics to improve gut health and treat microbiota-related conditions underscores the importance of ongoing research into bacterial strains [36,37,38,39]. The probiotic strains selected for the present study, *Lactobacillus rhamnosus* IMC 501^®^ and *Lactobacillus paracasei* IMC 502^®^, have been previously characterized in preclinical and human studies. Their combined administration has been shown to be well tolerated and biologically active in the human gastrointestinal tract, with reported effects on intestinal microbiota composition and bowel habits in healthy adults [40,41].

Although these observations derive from populations different from patients receiving home EN, they provide a rationale for exploring their potential effects in frail older adults affected by multimorbidity and functional decline. Accordingly, the present study evaluated whether supplementation of standard oligomeric EN with these strains was associated with changes in gut microbiota composition compared with conventional EN alone [42].

## 2. Materials and Methods

### 2.1. Participants and Study Protocol

This was a randomized open label, two-arm, parallel study conducted over a 30-day period designed to evaluate the effect of probiotic administration on the composition of microbiota in frail older patients with HEN, using an oligomeric mixture.

**Figure 1:**
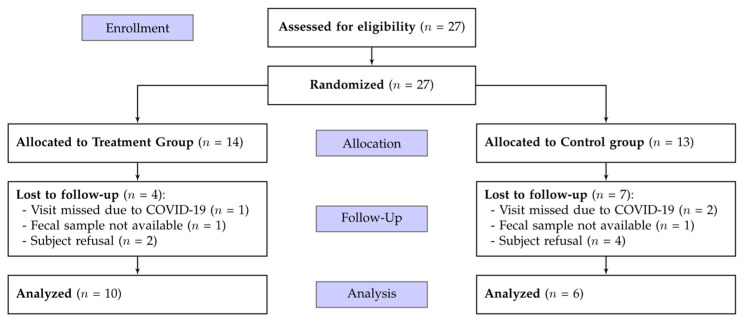
Schematic representation of the study workflow.

Participants were screened according to predefined inclusion and exclusion criteria and consecutively recruited from the Clinical Nutrition Unit of IRCCS INRCA (Ancona, Italy) between June 2018 and July 2024. Eligible participants were aged ≥ 65 years, had been receiving the same oligomeric enteral formula for at least one month prior to enrolment, and had no clinical diagnosis of inflammatory intestinal disease. Patients meeting enrollment criteria were randomized into two groups using a computer-generated allocation sequence following their order of inclusion. Participants who withdrew after receiving at least one dose of the study product were not replaced. Sixteen patients with comorbidities receiving EN with a di- and tripeptide-based oligomeric formula completed microbiota sampling at both time points and were included in the analysis. A participant flow diagram summarizing screening, allocation, follow-up, and analysis is shown in Figure 1.

The treatment group received probiotics at a dose of one 0.26 g capsule per day containing a 1:1 mixture of *Lactobacillus rhamnosus* IMC501^®^ and *Lactobacillus paracasei* IMC502^®^ (SYNBIO^®^, Synbiotec Srl, Camerino, Italy) for 30 days. The control group took only the oligomeric mixture for 30 days. Each capsule contained approximately 15 billion of live probiotic cells. These bacterial strains are both deposited in the German Collection of Microorganisms and Cell Cultures (DSMZ) and are protected by international patents (RM2004A000166 and EP 1743042). They are also officially registered with the Italian Ministry of Health (No. 49355). For EN, the probiotic powder was suspended into 5–10 mL of water and administered directly through the feeding tube.

All participants had been receiving peptide-based EN using standard oligomeric formulations based on di- and tripeptides commonly used in clinical practice. These formulas typically provide approximately 1.0 kcal/mL, with 4.0 g/100 mL of proteins mainly in the form of hydrolyzed peptides (~16% of total energy), 1.7 g/100 mL of lipids (~15% of total energy), and 17.6 g/100 mL of carbohydrates (~69% of total energy), without added dietary fiber. The EN regimen remained unchanged throughout the 30-day study period, with no modifications in formula type or dosage during the intervention phase.

Participants were excluded if they had used antibiotics or any probiotic not specified by the study protocol within one month prior to enrolment, had participated in another clinical trial that could potentially influence the intestinal microbiota, had a known hypersensitivity to any component of the probiotic formulation, were receiving partial EN with concurrent oral food intake (even in minimal amounts), or had any condition likely to impair compliance with the study protocol or otherwise contraindicate inclusion in the trial.

Fecal samples were collected from each participant at baseline and after 30 days of HEN, either with (treatment group) or without (control group) probiotic supplementation. During the observation period, intestinal motility and the incidence of *Clostridioides difficile* infection were monitored as clinical parameters.

The study was approved by the Institutional Review Board (or Ethics Committee) IRCCS INRCA (protocol code CE INRCA 17028, approval date 22 February 2018) and conducted in accordance with the Declaration of Helsinki. Informed consent was obtained from all participants. The study has been registered at ClinicalTrials.gov (Identifier: NCT07437352).

### 2.2. DNA Extraction and 16S rRNA Gene Amplicon Sequencing

Approximately 5 g of fecal matter was collected from each patient at baseline and at 30-day follow-up. This was placed in test tubes for stool storage with the help of nursing staff and/or caregivers. The stool samples were frozen at −80 °C until analysis by NGS.

Microbial DNA was extracted from faecal samples (one at baseline and one at 30-day follow-up) using the QIAamp PowerFecal Pro DNA Kit (Qiagen GmbH, Hilden, Germany). Amplification and sequencing were conducted in accordance with the manufacturer’s protocol; in particular, amplicons were generated using the Ion 16S™ Metagenomics Kit (Thermo Fisher Scientific, Waltham, MA, USA) with 3 ng of microbial DNA. This kit targets six hypervariable regions (V2, V4, V8, V3, V6–7 and V9) of the 16S rRNA gene using specific primers. The amplification conditions and subsequent procedures up to the end-repair phase were performed as previously described by Francini et al. [43]. All purification steps were carried out using MagSi-DNA beads (Magtivio, Nuth, The Netherlands) and the eluted products were resuspended in 20 µL of 1× low EDTA TE buffer (10 mM Tris base, 0.1 mM EDTA), pH 8.0. Equal volumes of the individual barcoded libraries were then pooled and diluted to a final concentration of 40 pM. Template preparation and chip loading were carried out using the Ion Chef system in accordance with the Ion 510, Ion 520 and Ion 530 Kit-Chef protocols. Sequencing was performed on the Thermo Fisher Scientific GeneStudio S5 system using a 400 bp run with a 520 chip. Base calling and run demultiplexing were performed using Torrent Suite version 5.18.1 with the default settings. Demultiplexed FASTQ files for each sample were generated using FileExporter version 5.12.0.0 (Thermo Fisher Scientific, Waltham, MA, USA). Ion Reporter (version 5.20.2.0) was used for data analysis with the “Metagenomics 16Sw1.1 v. 5.18” workflow and default parameters. The unaligned binary data files (BAM files) generated by Torrent Suite were uploaded to Ion Reporter for analysis. An average sequencing depth of 200,000 reads per sample was achieved.

### 2.3. Statistical Analysis

Continuous variables are presented as mean ± SD or median (IQR) based on normality assessment, categorical variables as frequencies, fractions, or percentages. Baseline characteristics were compared between treatment and control groups. Parametric and non-parametric tests were applied as appropriate for continuous variables, with Fisher’s exact tests for categorical variables. Between-group comparisons of alpha diversity were performed using the Wilcoxon rank-sum test, and within-group longitudinal changes between baseline and 30-day follow-up were evaluated using the Wilcoxon signed-rank test. Effect sizes are reported as Hodges–Lehmann location-shift estimates (Δ_HL_) with 95% confidence intervals. Microbiota abundance tables at genus and family level were analyzed without applying filtering thresholds. Alpha diversity indices (Shannon, Simpson, and Chao1) were calculated using the vegan R package. Beta diversity was analyzed via PERMANOVA on Bray–Curtis distances. PCoA was computed using classical multidimensional scaling on distance matrices, with separate analyses performed for each timepoint and taxonomic level. Given the limited sample size, a leave-one-out (LOO) sensitivity analysis was performed to evaluate the stability of the between-group comparisons at 30-day follow-up by iteratively repeating the analyses after excluding one subject at a time.

Statistical significance was set at *p* < 0.05; nominal *p*-values are reported, and Holm-adjusted *p*-values are provided as a sensitivity analysis for primary between-group comparisons at 30-day follow-up (R version 4.3.0).

## 3. Results

### 3.1. Population Characteristics

Sixteen frail older patients were enrolled, with a mean age of 81.4 ± 7.9 years and a BMI of 21.0 ± 3.7 kg/m^2^. The majority were female (81.2%), 43.8% resided in nursing homes, and 50.0% suffered from chronic constipation (Table 1). Cognitive decline was frequent, with 43.8% diagnosed with Alzheimer’s disease and 25.0% with dementia. Overall, no statistically significant differences were found between the control and treatment groups. All subjects were exclusively fed via HEN using the same type of commercial oligomeric formula, ensuring a standardized dietary intake across the entire cohort. According to the study protocol, antibiotic therapy within one month prior to enrolment was an exclusion criterion, and no antibiotic treatments were initiated during the 30-day follow-up period. Exposure to medications known to influence gut microbiota composition—limited in this cohort to proton pump inhibitors (PPIs) and osmotic laxatives—did not differ significantly between the two groups (*p* = 0.500 and *p* = 1.000, respectively), and these therapies remained stable both prior to enrolment and throughout the study period. Following the intervention, no significant changes were observed in the monitored clinical parameters, including intestinal motility and the incidence of *Clostridioides difficile* infection, in either the probiotic-supplemented group or the control group.

**Figure 2:**
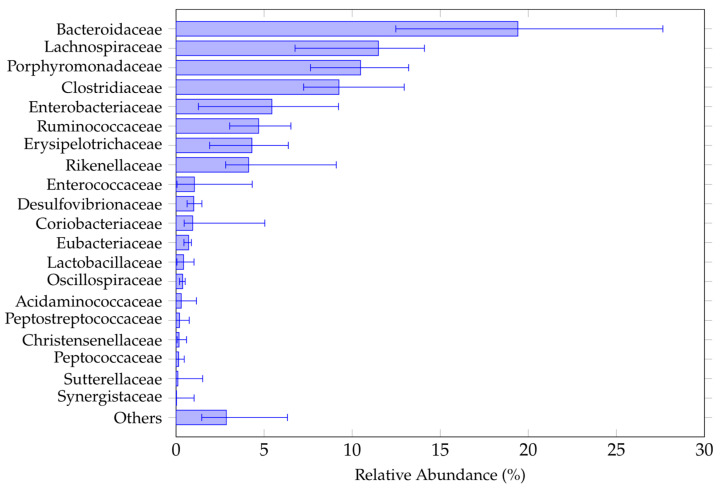
Relative abundances of the top 20 bacterial families in the gut microbiota of the study population at baseline. Bars represent median values, and whiskers indicate interquartile ranges (IQR). Families with lower relative abundance were grouped under the “others” category.

Figure 2 shows the twenty most abundant bacterial families identified in the gut microbiota of the study population at baseline. Although some variability between individuals was observed, the Bacteroidaceae family predominated, which is consistent with previous findings in frail older adults [16,44]. The full list of families identified in our study is provided in Table A1 (Appendix A.1).

### 3.2. Effects of Probiotic Supplementation on Alpha Diversity

**Figure 3:**
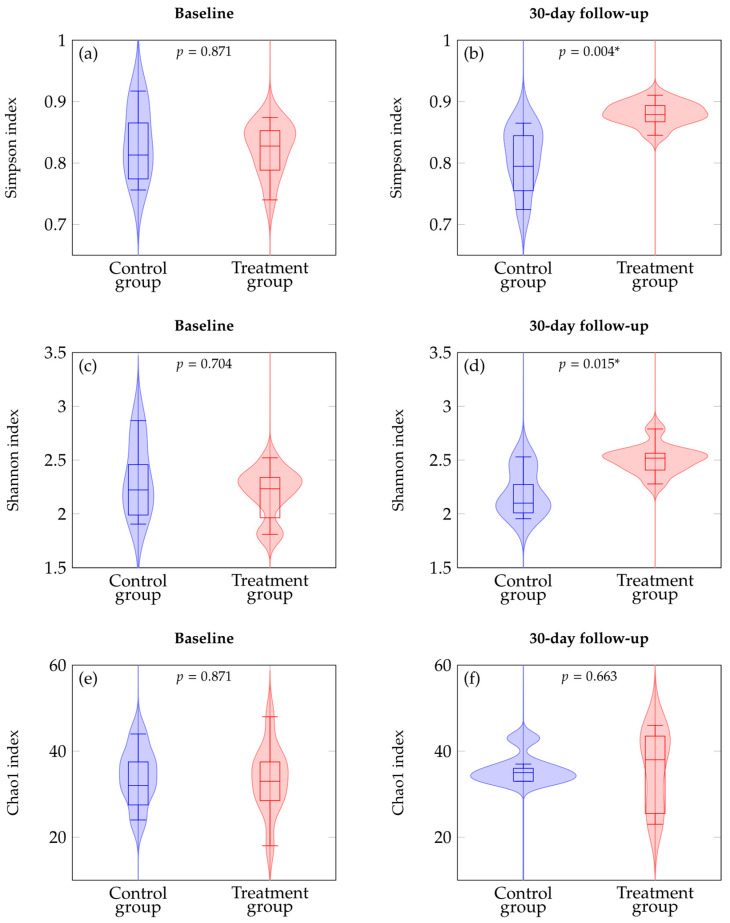
Alpha diversity analysis of gut microbiota at genus level. Violin plots show the distribution of Simpson diversity index (**a**,**b**), Shannon diversity index (**c**,**d**), and Chao1 richness estimator (**e**,**f**) comparing control and treatment groups at baseline and follow-up timepoints. Boxplots show median and quartiles. *p*-values from Mann–Whitney U tests are indicated above each comparison; asterisks (*) indicates statistically significant differences.

We used alpha and beta diversity indices to analyze the relationships between fecal microbiota samples at genus level. As shown in Figure 3, at baseline, no statistically significant differences were observed between treatment and control groups across all alpha diversity metrics. The Simpson diversity index was 0.827 (IQR 0.797–0.877) in controls and 0.838 (IQR 0.798–0.854) in treated patients (Δ_HL_ = +0.009; 95% CI −0.058 to +0.069; *p* = 0.871). The Shannon diversity index was 2.259 (IQR 2.110–2.539) in controls and 2.263 (IQR 2.130–2.340) in the treatment group (Δ_HL_ = +0.091; 95% CI −0.261 to +0.491; *p* = 0.704). Chao1 richness was 34.5 (IQR 31.3–37.8) in controls and 34.0 (IQR 29.3–37.8) in treated subjects (Δ_HL_ = +1.000; 95% CI −7.000 to +9.000; *p* = 0.871).

Conversely, 30-day follow-up analysis revealed significant differences in microbial alpha diversity. At the individual level, increases in both Simpson and Shannon diversity between baseline and follow-up were observed in 9/10 treated patients and in 2/6 controls. The Simpson index was 0.816 (IQR 0.788–0.848) in controls and 0.880 (IQR 0.871–0.894) in the treatment group (Δ_HL_ = −0.059; 95% CI −0.116 to −0.027; *p* = 0.004). The median within-subject change was Δ = −0.049 (IQR −0.079 to +0.043) in controls and Δ = +0.064 (IQR +0.020 to +0.078) in treated patients; paired individual-level analysis confirmed a significant increase in the treatment group (*p* = 0.008), whereas no significant change was observed in controls (*p* = 0.675). The Shannon index was 2.119 (IQR 2.072–2.340) in controls and 2.529 (IQR 2.425–2.565) in treated patients (Δ_HL_ = −0.354; 95% CI −0.503 to −0.039; *p* = 0.015). The median within-subject change was Δ = −0.157 (IQR −0.410 to +0.201) in controls and Δ = +0.282 (IQR +0.181 to +0.432) in treated patients; similarly, paired individual-level analysis indicated a significant increase in the treatment group (*p* = 0.008), whereas no significant change was detected in controls (*p* = 0.675). Chao1 richness was 35.0 (IQR 33.5–36.5) in controls and 39.5 (IQR 27.8–43.8) in the treatment group (Δ_HL_ = −2.254; 95% CI −9.000 to +10.000; *p* = 0.663). In contrast, increases in Chao1 richness between baseline and follow-up were observed in 3/6 controls and 6/10 treated patients. The median within-subject change was Δ = +1.5 (IQR −4.25 to +9.50) in controls and Δ = +7.5 (IQR −0.75 to +11.75) in treated patients; paired individual-level analysis did not show significant changes in either group (controls *p* = 0.673; treatment *p* = 0.406).

The between-group differences in alpha diversity at 30-day follow-up remained statistically significant after adjustment for multiple testing using the Holm method (Simpson p_adj_ = 0.012; Shannon p_adj_ = 0.030), while Chao1 richness remained non-significant (p_adj_ = 0.663). Given the small sample size, the stability of these findings was further assessed using LOO sensitivity analysis. Across all iterations, the between-group differences at 30-day follow-up remained statistically significant for both Shannon (LOO *p* range: 0.0059–0.0321) and Simpson diversity (LOO *p* range: 0.0027–0.0085), whereas Chao1 richness remained non-significant (LOO *p* range: 0.4064–0.9057), suggesting that the observed differences were unlikely to be driven by individual subjects. Within the constraints of the current dataset, additional exploratory analyses were conducted to further assess the stability of the observed patterns, including linear mixed-effects models accounting for repeated measurements, the results of which are reported in Appendix A.2.

### 3.3. Beta Diversity Shifts Following Probiotic Supplementation

**Figure 4:**
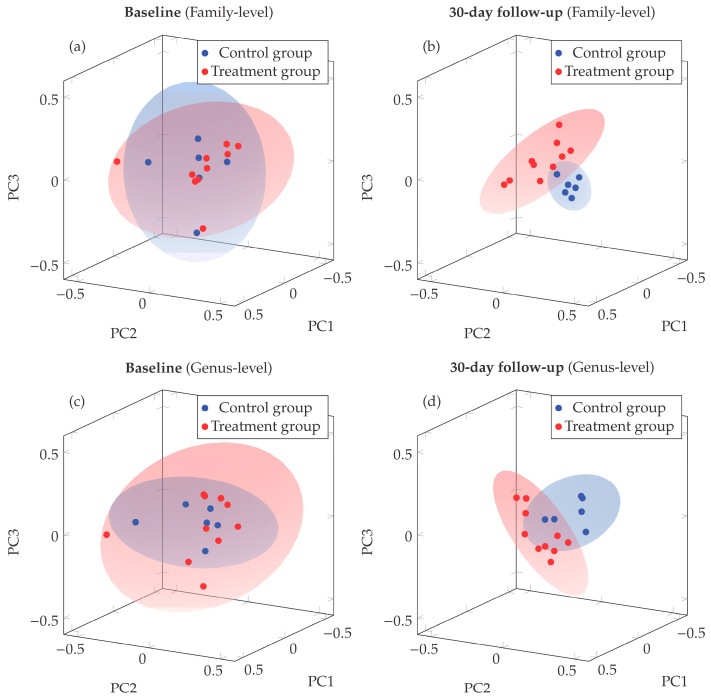
Bray–Curtis dissimilarity matrices at baseline (**a**,**c**) and 30-day follow-up (**b**,**d**) for both family-level (**a**,**b**) and genus-level (**c**,**d**) taxonomic resolution. Ellipsoids indicate 95% confidence intervals for group centroids. At family-level, the first three principal coordinates explained 73.3% (PC1: 38.4%, PC2: 18.7%, PC3: 16.2%) and 62.3% (PC1: 26.8%, PC2: 20.6%, PC3: 14.9%) of total variance at baseline and follow-up, respectively. At genus level, explained variance was 77.5% (PC1: 44.0%, PC2: 20.9%, PC3: 12.6%) and 58.0% (PC1: 22.9%, PC2: 18.8%, PC3: 16.3%) for baseline and follow-up, respectively. Sample sizes: *n* = 6 control, *n* = 10 treated.

Beta diversity was evaluated using a principal coordinate analysis (PCoA) based on Bray–Curtis distances, comparing samples collected at baseline and at the 30-day follow-up. As shown in Figure 4, the first three principal coordinates (PC1, PC2, and PC3) explained most of the variance in the dataset. At baseline, both family- and genus-level analyses displayed substantial overlap between treatment groups (Figure 4a,c), suggesting no major dissimilarities in the microbial community structure prior to the intervention.

In contrast, at 30-day follow-up, spatial separation emerged between control and treatment groups at both family and genus taxonomic levels. The primary drivers of community separation were identified through correlation analysis with principal coordinate axes (Table 2). At the family level, separation along PC3 was driven by relative enrichment of Lachnospiraceae and Erysipelotrichaceae in treated patients, versus Porphyromonadaceae and Sutterellaceae in controls. At the genus level, we found positive correlations with *Eubacterium* (PC3) and *Bifidobacterium* (PC1) in treated patients were found, while control patients showed stronger associations with *Bacteroides* and *Clostridium* (PC1) and *Sutterella* (PC3). These patterns highlight selective taxonomic shifts following probiotic supplementation.

This separation was particularly evident at the family level (Figure 4b), where participants who had not received probiotics (control group) clustered tightly within the positive PC2 and negative PC3 regions, whereas those who had taken probiotics (treatment group) showed a broader dispersion across the positive PC1 and PC3, and negative PC2 dimensions. A similar pattern was observed at the genus level (Figure 4d), where the control group clustered within the negative PC1 and positive PC2 areas, whereas treatment group displayed a broader distribution across the positive PC1 and negative PC2 and PC3 axes.

These broader dispersions among treated individuals, which are consistent across both taxonomic levels, may be explained in terms of higher interindividual variability in microbial composition following the nutritional intervention compared with the control group. A more detailed characterization of this phenomenon revealed coordinated variation between principal coordinates in treated subjects at the 30-day follow-up. At the family level, treated subjects exhibited significant correlations between PC1 and PC3 (R = −0.718, *p* = 0.019) and between PC2 and PC3 (R = 0.652, *p* = 0.041). At the genus level, treated subjects displayed correlation between PC2 and PC3 (R = −0.737, *p* = 0.015). These correlations, highlighted by an oblique dispersion of data clouds in PCoA space, suggest that taxa loading on respective principal coordinates tends to vary in a coordinated manner within the treatment group, rather than a uniform shift affecting all individuals equally.

Differences in microbial community composition were assessed using PERMANOVA at both the genus and family taxonomic levels. At baseline, no significant separation was observed between control and treated patients at either genus (PERMANOVA: F = 0.66, R^2^ = 0.045, *p* = 0.67) and family levels (PERMANOVA: F = 0.59, R^2^ = 0.040, *p* = 0.77). In contrast, at 30-day follow-up, significant divergence in community structure was confirmed at both genus (PERMANOVA: F = 2.27, R^2^ = 0.139, *p* = 0.008) and family levels (PERMANOVA: F = 2.45, R^2^ = 0.149, *p* = 0.011). At follow-up, the observed differences in community structure at both genus and family levels remained statistically significant after adjustment for multiple testing using the Holm method (p_adj_ = 0.016 for both taxonomic levels). This multi-level taxonomic consistency strengthens the evidence that the nutritional intervention induced measurable changes in gut microbiota structure, with treated subjects developing distinct microbial profiles compared to controls regardless of taxonomic resolution examined. Longer follow-up studies will be required to determine whether these changes translate into stable microbiota configurations over time.

## 4. Discussion

This study investigated whether supplementing the diets of frail older patients receiving EN with probiotics containing *Lactobacillus rhamnosus* and *Lactobacillus paracasei* could alter the composition of their gut microbiota. The study population was characterized by advanced age, polypharmacy, cognitive decline, and nutritional vulnerability—a clinically complex group that is rarely considered in microbiota research. In fact, only a few studies have considered the composition of the gut microbiota of older patients with a relatively long history of EN [45,46]. In our study we found that the microbiota of patients on an oligomeric diet was particularly enriched, at family level, of Bacteroidaceae, Lachnospiraceae, Pophyromonadaceae and Clostridiaceae, among others. The presence of Lachnospiraceae, which are the main producers of SCFAs, could explain the need to add specific substrates to the enteral formula [47]. The Bacteroideceae and Porphyromonadaceae families belong to the Bacteroidetes phylum and are typically found in older individuals [16].

Clostridiaceae present a complex scenario, as this family comprises a wide range of phylogenetic and functional heterogeneity. It encompasses both beneficial bacteria, some of which produce butyrate, and potentially pathogenic species, such as *Clostridium difficile* [48]. Aging increases susceptibility to opportunistic pathogens, notably Clostridioides difficile, which contributes to intestinal inflammation, nutritional deficits, and reduced Bifidobacteria. Evidence indicates that probiotic supplementation can lower the risk of *C. difficile*-related complications in elderly surgical patients [49].

Alpha diversity analysis showed no significant baseline differences between treated and control groups, consistent with the long-term administration of a uniform oligomeric EN formula prior to the intervention [9,10,27].

An increase in alpha diversity, as measured by the Shannon and Simpson indices, was observed in the treated group compared to the control group at 30-day follow-up, indicating an increase in overall microbial diversity. Previous studies have shown that a greater diversity of gut microbes is associated with improved intestinal health and greater resilience in healthy older individuals. A greater microbial diversity is also associated with increased metabolic activity of the microbiota and more effective immune responses [47,48,50]. Therefore, the increase in biodiversity observed in the treated group after 30 days of probiotic supplementation represents a shift toward a more diverse microbial configuration, which is a feature generally associated with gut ecosystem resilience [49].

Bray–Curtis PCoA and PERMANOVA confirmed that, at baseline, control and treated subjects exhibited comparable microbial community structures. After 30 days, a statistically significant separation between groups (*p* < 0.011) emerged, accompanied by a broader dispersion of the treatment group in ordination space, indicating increased heterogeneity in community composition compared with the control group. The distinctive orientation and dispersion pattern further suggest a structured reorganization of microbial community composition in the treatment group. Specifically, the increased diversity observed in treated subjects reflects coordinated variation among taxa, where relative enrichment in certain groups (e.g., *Lachnospiraceae*) tends to coincide with depletion in others (e.g., *Ruminococcaceae*), consistent with a shift toward a more heterogeneous microbial configuration compared with the pattern observed in controls.

Beta diversity PCoA ordination of Bray–Curtis analysis can identify the bacterial families and genera responsible for this change. At the family level, this change was associated with an increase in the relative abundance of Lachnospiraceae and Erysipelotrichaceae in patients who received treatment. These two families belong to the Firmicutes phylum and are characterized by their saccharolytic capacities and their ability to efficiently convert dietary substrates and host-derived glycans into short-chain fatty acids (SCFAs), such as butyrate and propionate [51].

At the genus level, positive correlations in patients treated with probiotics included *Eubacterium*, and *Bifidobacterium*. Eubacterium species are well-recognized butyrate producers and are a key component of intestinal trophic chains. They contribute to the conversion of lactate and acetate—metabolites frequently derived from Bifidobacterium fermentation—into butyrate. Butyrate is a major energy source for colonocytes and is crucial for maintaining gut barrier integrity and immune regulation [52,53].

Persistence or permanent colonization of the administered probiotic strains cannot be determined in the present study, as strain-specific tracking methods (e.g., qPCR or strain-resolved sequencing) were not performed. Nevertheless, it is reasonable to hypothesize that the administered probiotics may have transiently influenced the composition and ecological interactions of the resident gut microbiota through metabolic or immunomodulatory mechanisms [54].

Finally, we observed that the gut microbiota of control patients showed stronger associations with *Bacteroides*, *Clostridium*, and *Sutterella*. *Bacteroides* (phylum Bacteroidetes) and *Sutterella* (phylum Proteobacteria) are often associated with intestinal dysbiosis and low-grade inflammation, particularly in older adults. Excessive abundance of *Bacteroides* may compromise gut barrier integrity and increase pro-inflammatory metabolite production, while expansion of *Sutterella* reflects altered microbial balance and is linked to chronic intestinal and systemic inflammatory states. Together, their enrichment has been described as a microbial signature of impaired gut homeostasis in frail elderly populations [21,55,56,57,58,59].

Furthermore, in addition to having a higher presence of Proteobacteria, frail individuals may have an intestinal microbiota with a prevalence of opportunistic bacteria, such as certain Clostridium species [48].

## 5. Conclusions and Limitations

This randomized, open-label, two-arm parallel study showed that short-term supplementation with *Lactobacillus rhamnosus* and *Lactobacillus paracasei* was associated with measurable changes in gut microbiota composition and diversity in a small cohort of older patients receiving home EN. These changes were primarily reflected in differences in alpha diversity metrics and overall community structure during the intervention period.

The present findings should be interpreted in the context of several limitations. First, the relatively small sample size reflects the exploratory nature of this pilot study and limits the statistical power to detect consistent microbiota changes in a population characterized by high inter-individual variability. Second, the study focused primarily on taxonomic composition and did not include functional measurements such as inflammatory biomarkers, microbial metabolites, or gut barrier markers. Consequently, the biological and clinical implications of the observed microbiota patterns remain uncertain. Third, the 30-day intervention period does not allow conclusions regarding the persistence or long-term stability of microbiota changes. Finally, the clinical complexity of frail older adults receiving HEN (multimorbidity, polypharmacy, and variable duration of enteral feeding) may contribute to heterogeneity in microbiota composition.

Consequently, statistical power and generalizability are limited, and the clinical relevance of the observed microbiota changes remains to be established.

The clinical complexity typical of this patient population may contribute to inter-individual variability in baseline gut microbiota composition, a feature commonly reported in studies involving frail older adults receiving long-term EN. Within this context, the present study was designed to describe short-term microbiota patterns associated with probiotic supplementation. Although the follow-up period was limited, the observed changes provide preliminary insights that may help guide future larger studies designed to evaluate longer-term ecological and functional effects. Therefore, these results should be considered exploratory and hypothesis-generating rather than definitive evidence of probiotic efficacy in this population.

As the analysis focused on microbiota structure, functional implications were inferred indirectly and should be interpreted in the context of the existing literature. While the observed variations in the relative abundance patterns of specific taxa were exploratory, they provide a preliminary basis to motivate future studies integrating metabolomic or multi-omic approaches to further investigate microbiota–host interactions in this clinically fragile and understudied population and to confirm these findings in larger, clinically stratified cohorts.

## Figures and Tables

**Table 1:** Baseline characteristics of the study population. Continuous variables are expressed as mean ± SD; dichotomous variables as frequencies and percentages.

	Total (*n* = 16)	Control Group (*n* = 6)	Treatment Group (*n* = 10)	*p*-Value
Age (years)	81.4 ± 7.9	83.3 ± 4.4	80.3 ± 9.4	0.398
Body mass index (kg/m^2^)	21.0 ± 3.7	22.6 ± 4.4	19.9 ± 3.0	0.218
N° medications	7.2 ± 3.9	5.6 ± 3.5	8.8 ± 3.9	0.210
Female	13 (81.2%)	6 (100.0%)	7 (70.0%)	0.250
Constipation	8 (50.0%)	2 (33.3%)	6 (60.0%)	0.608
Nursing home	7 (43.8%)	2 (33.3%)	5 (50.0%)	0.633

**Table 2:** Top 10 microbial families and genera that most strongly influence the ordination patterns observed in PCoA analysis. (Taxa are ranked by absolute correlation coefficients (|r|) with each principal coordinate axis).

PC1	PC2	PC3
Family-level Analysis:		
*Bacteroidaceae* (−0.907)	*Ruminococcaceae* (−0.821)	*Lachnospiraceae* (0.871)
*Corynebacteriaceae* (0.689)	*Catabacteriaceae* (−0.793)	*Erysipelotrichaceae* (0.789)
*Coriobacteriaceae* (0.606)	*Christensenellaceae* (−0.708)	*Sphingobacteriaceae* (0.769)
*Acidaminococcaceae* (−0.579)	*Bacillaceae* (−0.691)	*Oxalobacteraceae* (0.667)
*Bifidobacteriaceae* (0.565)	*Peptococcaceae* (−0.668)	*Streptococcaceae* (0.593)
*Prevotellaceae* (−0.538)	*Veillonellaceae* (0.641)	*Leuconostocaceae* (0.531)
*Clostridiaceae* (−0.529)	*Enterobacteriaceae* (0.615)	*Peptostreptococcaceae* (0.523)
*Staphylococcaceae* (0.507)	*Clostridiales Family XI* (0.613)	*Flavobacteriaceae* (0.473)
*Clostridiales Family XI* (0.486)	*Enterococcaceae* (−0.601)	*Porphyromonadaceae* (−0.471)
*Enterobacteriaceae* (0.434)	*Oscillospiraceae* (−0.583)	*Sutterellaceae* (−0.467)
Genus-level Analysis:		
*Bacteroides* (−0.778)	*Collinsella* (0.776)	*Eubacterium* (−0.82)
*Clostridium* (−0.624)	*Raoultella* (0.776)	*Alistipes* (−0.692)
*Bilophila* (0.589)	*Anaerococcus* (0.775)	*Sutterella* (0.624)
*Enterococcus* (0.575)	*Megamonas* (0.771)	*Herbaspirillum* (−0.615)
*Olsenella* (0.558)	*Marvinbryantia* (0.771)	*Oxalobacter* (−0.580)
*Bifidobacterium* (0.523)	*Dialister* (0.771)	*Anaerostipes* (−0.545)
*Corynebacterium* (0.517)	*Finegoldia* (0.749)	*Catabacter* (−0.507)
*Finegoldia* (0.498)	*Bifidobacterium* (0.729)	*Subdoligranulum* (0.497)
*Phascolarctobacterium* (−0.497)	*Olsenella* (0.710)	*Candidatus soleaferrea* (−0.484)
*Desulfovibrio* (−0.493)	*Eggerthella* (−0.582)	*Enorma* (0.456)

Values in parentheses represent Pearson correlation coefficients between taxa relative abundance and principal coordinate scores. Positive correlations indicate taxa with higher relative abundance in samples with higher PC scores, while negative correlations indicate association with lower PC scores. These contributory taxa represent the primary drivers of microbial community structure variation at follow-up, explaining the separation patterns between treatment groups observed in the three-dimensional ordination space. Analysis was performed at both family and genus taxonomic levels to provide complementary resolution of community drivers.

## Data Availability

The data supporting the findings of this study are available from the corresponding author upon reasonable request for research purposes, in accordance with applicable data protection regulations.

## Abbreviations

The following abbreviations are used in this manuscript:

HEN	Home Enteral Nutrition
EN	Enteral Nutrition
PEG	Percutaneous endoscopic gastrostomy
SCFAs	Short-chain fatty acids
SD	Standard deviation
IQR	Interquartile range
LOO	Leave-One-Out
BMI	Body mass index
PPI	Proton pump inhibitors
CI	Confidence intervals
CV	Cardiovascular
CNS	Central Nervous System
PC	Principal Coordinate
PCoA	Principal Coordinate Analysis

## Appendix A

### Appendix A.1

The table includes all monitored families to provide a comprehensive view of the taxonomic landscape at baseline. For clarity, taxa were ordered primarily by decreasing median relative abundance and secondarily by decreasing third-quartile values.

**Table A1:** Relative abundances at baseline for all detected taxa, expressed as percentages and reported as median values with their interquartile ranges.

Family	1st Quartile	Median	3rd Quartile
Bacteroidaceae	12.48%	19.41%	27.64%
Lachnospiraceae	6.76%	11.49%	14.10%
Porphyromonadaceae	7.64%	10.47%	13.21%
Clostridiaceae	7.24%	9.25%	12.96%
Enterobacteriaceae	1.27%	5.44%	9.23%
Ruminococcaceae	3.04%	4.69%	6.52%
Erysipelotrichaceae	1.91%	4.30%	6.37%
Rikenellaceae	2.82%	4.12%	9.10%
Enterococcaceae	0.05%	1.04%	4.32%
Desulfovibrionaceae	0.63%	1.00%	1.47%
Coriobacteriaceae	0.46%	0.94%	5.04%
Eubacteriaceae	0.45%	0.71%	0.87%
Lactobacillaceae	0.05%	0.43%	1.02%
Unclassified clostridiales	0.26%	0.39%	0.74%
Oscillospiraceae	0.19%	0.37%	0.52%
Acidaminococcaceae	0.00%	0.29%	1.16%
Peptostreptococcaceae	0.00%	0.19%	0.75%
Christensenellaceae	0.06%	0.17%	0.59%
Peptococcaceae	0.00%	0.14%	0.46%
Sutterellaceae	0.00%	0.10%	1.51%
Synergistaceae	0.00%	0.03%	1.02%
Prevotellaceae	0.00%	0.02%	0.33%
Pseudomonadaceae	0.00%	0.02%	0.09%
Streptococcaceae	0.00%	0.01%	0.23%
Victivallaceae	0.00%	0.01%	0.12%
Clostridiales family xiii incertae sedis	0.00%	0.00%	0.09%
Corynebacteriaceae	0.00%	0.00%	0.30%
Clostridiales family xi incertae sedis	0.00%	0.00%	0.03%
Bifidobacteriaceae	0.00%	0.00%	3.19%
Clostridiales family xii incertae sedis	0.00%	0.00%	0.07%
Gracilibacteraceae	0.00%	0.00%	0.07%
Verrucomicrobiaceae	0.00%	0.00%	0.03%
Bacillaceae	0.00%	0.00%	0.01%
Leuconostocaceae	0.00%	0.00%	0.01%
Sphingobacteriaceae	0.00%	0.00%	0.01%
Catabacteriaceae	0.00%	0.00%	0.00%
Thermoanaerobacterales family iv incertae sedis	0.00%	0.00%	0.00%
Beutenbergiaceae	0.00%	0.00%	0.00%
Oxalobacteraceae	0.00%	0.00%	0.00%
Actinomycetaceae	0.00%	0.00%	0.00%
Planococcaceae	0.00%	0.00%	0.00%
Carnobacteriaceae	0.00%	0.00%	0.00%
Veillonellaceae	0.00%	0.00%	0.00%
Thermoanaerobacterales family iii incertae sedis	0.00%	0.00%	0.00%
Nitrospinaceae	0.00%	0.00%	0.00%
Comamonadaceae	0.00%	0.00%	0.00%
Geobacteraceae	0.00%	0.00%	0.00%
Colwelliaceae	0.00%	0.00%	0.00%
Staphylococcaceae	0.00%	0.00%	0.00%
Oligosphaeraceae	0.00%	0.00%	0.00%
Propionibacteriaceae	0.00%	0.00%	0.00%
Micrococcaceae	0.00%	0.00%	0.00%
Peptoniphilaceae	0.00%	0.00%	0.00%
Brevibacteriaceae	0.00%	0.00%	0.00%
Alteromonadaceae	0.00%	0.00%	0.00%
Microbacteriaceae	0.00%	0.00%	0.00%
Acetobacteraceae	0.00%	0.00%	0.00%
Paenibacillaceae	0.00%	0.00%	0.00%
Moraxellaceae	0.00%	0.00%	0.00%
Campylobacteraceae	0.00%	0.00%	0.00%
Alcaligenaceae	0.00%	0.00%	0.00%
Flavobacteriaceae	0.00%	0.00%	0.00%
Cytophagaceae	0.00%	0.00%	0.00%
Brachyspiraceae	0.00%	0.00%	0.00%
Thermoanaerobacteraceae	0.00%	0.00%	0.00%
Defluviitaleaceae	0.00%	0.00%	0.00%
Halanaerobiaceae	0.00%	0.00%	0.00%
Syntrophomonadaceae	0.00%	0.00%	0.00%
Proteinivoraceae	0.00%	0.00%	0.00%

### Appendix A.2

To further explore the longitudinal structure of the data, in addition to the non-parametric analyses reported in this study, linear mixed-effects models including treatment, timepoint, and their interaction were fitted for each alpha diversity metric, with subject included as a random intercept to account for repeated measurements. These explorative results are summarized in Table A2.

**Table A2:** Linear mixed-effects models testing the treatment × timepoint interaction.

Metric	F (1, 28)	*p*-Value
Simpson	6.84	0.014
Shannon	6.64	0.016
Chao1	0.04	0.838
